# Correlative proteomics identify the key roles of stress tolerance strategies in *Acinetobacter baumannii* in response to polymyxin and human macrophages

**DOI:** 10.1371/journal.ppat.1010308

**Published:** 2022-03-01

**Authors:** Zhi Ying Kho, Mohammad A. K. Azad, Mei-Ling Han, Yan Zhu, Cheng Huang, Ralf B. Schittenhelm, Thomas Naderer, Tony Velkov, Joel Selkrig, Qi (Tony) Zhou, Jian Li

**Affiliations:** 1 Biomedicine Discovery Institute, Infection Program and Department of Microbiology, Monash University, Clayton, Victoria, Australia; 2 Monash Proteomics & Metabolomics Facility, Monash Biomedicine Discovery Institute, Monash University, Clayton, Victoria, Australia; 3 Biomedicine Discovery Institute, Infection Program, Department of Biochemistry and Molecular Biology, Monash University, Clayton, Victoria, Australia; 4 Department of Pharmacology and Therapeutics, School of Biomedical Sciences, Faculty of Medicine, Dentistry and Health Sciences, University of Melbourne, Parkville, Victoria, Australia; 5 European Molecular Biology Laboratory, Genome Biology Unit, Heidelberg, Germany; 6 Department of Industrial and Physical Pharmacy, Purdue University, West Lafayette, Indiana, United States of America; Washington State University, UNITED STATES

## Abstract

The opportunistic pathogen *Acinetobacter baumannii* possesses stress tolerance strategies against host innate immunity and antibiotic killing. However, how the host-pathogen-antibiotic interaction affects the overall molecular regulation of bacterial pathogenesis and host response remains unexplored. Here, we simultaneously investigate proteomic changes in *A*. *baumannii* and macrophages following infection in the absence or presence of the polymyxins. We discover that macrophages and polymyxins exhibit complementary effects to disarm several stress tolerance and survival strategies in *A*. *baumannii*, including oxidative stress resistance, copper tolerance, bacterial iron acquisition and stringent response regulation systems. Using the *spoT* mutant strains, we demonstrate that bacterial cells with defects in stringent response exhibit enhanced susceptibility to polymyxin killing and reduced survival in infected mice, compared to the wild-type strain. Together, our findings highlight that better understanding of host-pathogen-antibiotic interplay is critical for optimization of antibiotic use in patients and the discovery of new antimicrobial strategy to tackle multidrug-resistant bacterial infections.

## Author summary

Bacterial response towards antibiotics is sensitive to the surrounding environment; however, how the host immune microenvironment affects the response of opportunistic pathogen *Acinetobacter baumannii* towards polymyxins remains largely unexplored. In this study, we established a host-pathogen-antibiotic tripartite *in vitro* model to examine the complex tripartite molecular interplay from both bacteria and macrophages perspectives, mimicking the physiological infection-treatment condition. This is the first comprehensive proteomics dataset for *A*. *baumannii* interacting with host cells, with an extraordinary coverage (i.e., 50%) of *A*. *baumannii* proteome even in infection conditions. For the first time, we report that macrophages and polymyxins utilize complementary mechanisms to disarm several *A*. *baumannii* stress tolerance strategies to persist in the macrophages. Our host-pathogen-antibiotic mechanistic study and the discovery of potential druggable targets (e.g., bacterial stringent stress response regulator SpoT) represent a significant advance, paving way to antibiotic optimization and drug discovery to tackle multidrug-resistant bacterial infections.

## Introduction

Antimicrobial resistance (AMR) has been recognized as a major global threat with severe health and economic consequences [1,2]. One of the most important emerging pathogens is multidrug-resistant (MDR) *Acinetobacter baumannii* which causes severe pneumonia, bacteremia and other infections, especially in immunocompromised patients [3]. Indeed, carbapenem-resistant *A*. *baumannii* has been highlighted by the World Health Organization (WHO) as one of the three top-priority pathogens (Priority 1: Critical) urgently requiring the discovery and development of novel antibiotics [4]. Unfortunately, the increasing emergence and spread of antibiotic resistance has thus far outcompeted the discovery of novel antibiotics [5,6]. Notwithstanding the recently approved siderophore cephalosporin cefiderocol to treat extensively drug-resistant Gram-negative infections [7], the ‘old’ polymyxins (i.e., polymyxin B and colistin) are often the only therapeutic option for otherwise untreatable bacterial infections, including those caused by *A*. *baumannii* [8,9]. Polymyxins are membrane-targeting cyclic lipopeptide antibiotics that are active against many of the MDR Gram-negative bacterial pathogens responsible for nosocomial infections, such as *A*. *baumannii* [10–15]. However, polymyxin resistance has been increasingly reported since their re-introduction to the clinic in the early 2000s, with sub-optimal use as a major contributor [16,17]. Therefore, optimization of the dosage regimens of polymyxins is urgently required to maximize the antibacterial efficacy, reduce the emergence of resistance and preserve their clinical utility.

Current antibiotic pharmacokinetics/pharmacodynamics (PK/PD) are significantly limited by a lack of understanding of the complex interplay between the host, pathogen and drug [9,18]. Most studies emphasize either pathogen-drug, host-pathogen or host-drug bipartite interactions at any one time, neglecting the effect of a dynamic host environment which includes modulation of antibiotic activity against bacteria by the innate immune system. As *A*. *baumannii* is an opportunistic pathogen, better understanding of its interaction with host innate immune cells is critical for the optimization of antibiotic treatment strategies [19,20]. In this regard, macrophages are a critical component of early host responses to invading bacteria, capable of phagocytosing a low number of bacteria prior to the subsequent recruitment of neutrophils [21]. *In vivo* ablation of alveolar macrophages has also been shown to exacerbate bacterial burdens in mice infected by *A*. *baumannii* [21,22]. In *A*. *baumannii*, several virulence and immune evasion factors have been identified which assist the pathogen to evade neutrophil chemotaxis, including the cytotoxic outer membrane protein A (OmpA), immunogenic lipopolysaccharide, phagocytosis-preventing capsular polysaccharide, and the phenylacetic acid catabolic pathway [23–26]. The specific bacterial machineries that facilitate *A*. *baumannii* persistence in macrophages remain to be elucidated. Understanding the correlative global protein expression profiles of both the host and pathogen is critical for the identification of key biochemical pathways and protein targets underlying the host-pathogen interaction [27]. To the best of our knowledge, no study has yet examined the molecular interactions between macrophages and interacting (adherent and intracellular) *A*. *baumannii* in the presence of antibiotics, limiting our understanding on how the unique macrophage microenvironment affects the bacterial response. Technical challenges exist particularly in sufficient coverage of bacterial proteins for proteomic analysis upon interaction with host cells [27].

Here we employed proteomics to elucidate the complex interplay among MDR *A*. *baumannii*, human macrophages and polymyxins. Using differentiated THP-1 human macrophages (THP-1-dMs) and the model MDR isolate *A*. *baumannii* AB5075, we simultaneously investigated proteome changes of both the pathogen and host in the absence or presence of the polymyxins. Interacting bacterial fraction was focused in this study and sufficient numbers of bacterial cells were obtained for proteomic analysis by a two-step differential centrifugation method. With 1,954 bacterial proteins and 4,320 mammalian proteins identified, we discovered the unique proteome signatures that underlie macrophage immune-inflammatory responses following infection, and *A*. *baumannii* tolerance strategies in the presence of macrophage and polymyxin. Finally, an *A*. *baumannii* transposon mutant library was employed to demonstrate that impairment of bacterial redox stress resistance, iron acquisition and stringent response regulators significantly enhanced bacterial killing by polymyxin. Our findings provide critical mechanistic information for optimizing polymyxin use in patients.

## Results

### Proteomic profiling of *A*. *baumannii*

We examined whether macrophages (THP-1-dMs) alter molecular response of *A*. *baumannii* AB5075 towards 30 mg/L polymyxin B (PMB) treatment or vice versa, with a focus on the interacting bacterial fraction (**S1 Fig**). A total of 1,954 proteins were identified across all bacteria-associated samples that covered approximately 50% of the AB5075 proteome. To the best of our knowledge, this is the first comprehensive proteomics dataset for *A*. *baumannii* interacting with host cells. The principal component analysis (PCA) revealed that the expression data of bacteria treated with polymyxin B only (*A*. *baumannii* [AB] + PMB) exhibited the most distinct separation from untreated bacterial controls in the first component, followed by the polymyxin B-treated infection group (AB + THP-1-dMs + PMB) and THP-1-dMs infection alone group (AB + THP-1-dMs) (**S2A Fig**). Compared to the untreated bacterial controls, AB + THP-1-dMs + PMB resulted in 607 differentially expressed proteins (DEPs) followed by AB + PMB (549 DEPs) and AB + THP-1-dMs (202 DEPs), indicating that perturbations in the AB5075 proteome were predominantly driven by polymyxin treatment (**S2B Fig**).

#### THP-1-dMs induced modulation of redox and iron metabolism in *A. baumannii*

**Fig 1:**
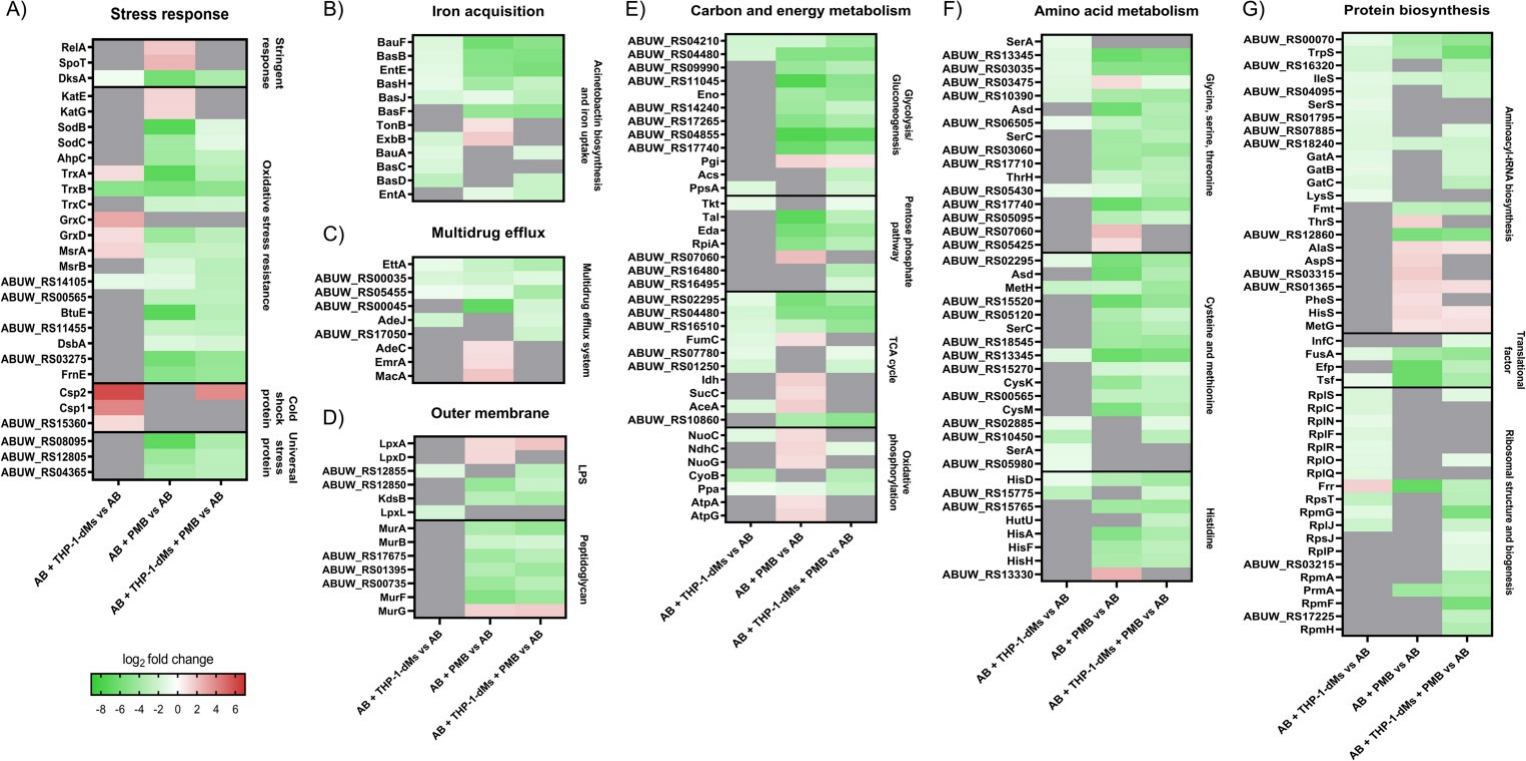
Polymyxins and THP-1-dMs cause unique proteomic changes in *A*. *baumannii*. Heatmaps showing protein expression changes of AB5075 in **A**) stress response; **B**) metal acquisition; **C**) multidrug efflux; **D**) outer membrane; **E**) carbon and energy metabolism; **F**) amino acid metabolism; and **G**) protein biosynthesis in respective comparison groups. The colour code shows log_2_FC values of differentially expressed proteins with FDR <0.05 compared to that of untreated controls. Grey indicates protein expression exceeded an FDR of 0.05. Data are presented as mean of three biological replicates per experimental group. AB, wild-type *A. baumannii* AB5075; PMB, polymyxin B; THP-1-dMs, macrophage-like THP-1 cells.

**Fig 2:**
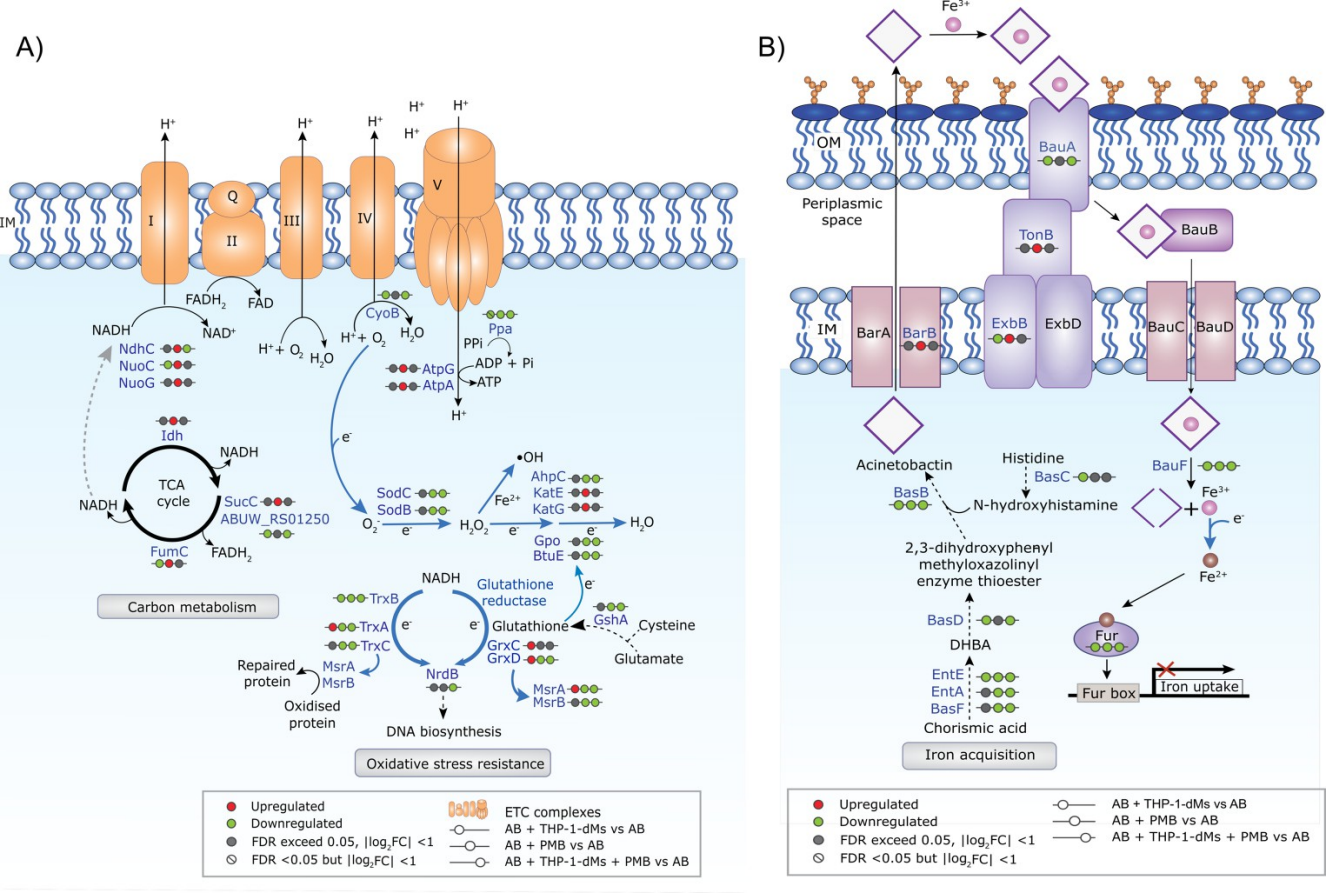
*A*. *baumannii* differentially remodels oxidative stress resistance and iron homeostasis following different treatments. Schematic diagram illustrating protein networks in AB5075 associated with **A**) carbon metabolism and oxidative stress resistance, and **B**) acinetobactin biosynthesis and iron acquisition. Red and green indicate upregulated and downregulated differentially expressed proteins, respectively. Data are presented as mean of three biological replicates per experimental group. IM, inner membrane; OM, outer membrane; ETC, electron transport chain; Gpo, glutathione peroxidase (ABUW_RS11455); GshA, glutamate—cysteine ligase (ABUW_RS00565); NrdB, ribonucleotide-diphosphate reductase subunit beta (ABUW_RS15470); DHBA, 2,3-dihydroxybenzoic acid; EntA, SDR family oxidoreductase (ABUW_RS10080).

In response to THP-1-dMs, interacting bacteria uniquely upregulated several cold shock proteins namely Csp2 (ABUW_RS12225), Csp1 (ABUW_RS13055) and putative cold shock protein (ABUW_RS15360) with respective log_2_FC values of 6.12, 4.12 and 1.23, compared to the untreated controls (**Fig 1A**). Interacting bacteria also upregulated oxidative stress resistance-associated thioredoxin TrxA, glutaredoxins GrxC and GrxD, peptide-methionine (S)-S-oxide reductase MsrA, and an oxidative damage protection protein (ABUW_RS17615) (**Fig 1A and S1 Table**). THP-1-dMs infection alone also led to downregulation in bacterial iron acquisition systems. Several acinetobactin biosynthetic enzymes were significantly downregulated in the AB + THP-1-dMs group, including 2,3-dihydroxybenzoate-AMP ligase EntE, non-ribosomal peptide synthetase BasD, BasC and BasB, TonB-dependent siderophore receptors BauA, TonB-dependent receptor (ABUW_RS14495), FhuE (ABUW_RS08070), MotA/TolQ/ExbB proton channel family protein (ExbB, ABUW_RS16650), and acinetobactin utilization protein BauF (**Fig 1B**). **Fig 2A** and **2B** show the schematics of oxidative stress resistance and iron acquisition pathways, respectively, with up- and down-regulated DEPs.

#### Polymyxin B perturbed bacterial redox, energy, iron homeostasis and stringent response in *A. baumannii*

The bacterial proteome signature corresponding to antibiotic treatment alone (i.e., the AB + PMB group) includes a unique upregulation of catalases KatE and KatG, and severe downregulation of superoxide dismutases SodB (ABUW_RS05940) and SodC (ABUW_RS01670), with log_2_FC values of 1.46, 1.37, -7.32 and -4.08, respectively (**Figs 1A and 2A**). Notably, two thioredoxins (TrxA and TrxC), thioredoxin-disulfide reductase (TrxB), GrxD, and glutathione peroxidases BtuE (ABUW_RS18150) were substantially downregulated (log_2_FC values of -7.20, -2.23, -5.73, -4.31, and -7.21, respectively) (**Figs 1A and 2A**). Furthermore, polymyxin B treatment alone downregulated AB5075 protein quality control systems, including the DsbC family protein (ABUW_RS03275), DsbA family oxidoreductase (FrnE, ABUW_RS17415), thiol:disulfide interchange protein DsbA/DsbL (DsbA, ABUW_RS18725), MsrA and peptide-methionine (R)-S-oxide reductase MsrB (**Fig 1A**).

Interestingly, we observed a unique upregulation in tricarboxylic acid (TCA) cycle and oxidative phosphorylation proteins with polymyxin B treatment alone. These upregulated proteins included NADP-dependent isocitrate dehydrogenase Idh (ABUW_RS05265), ADP-forming succinate-CoA ligase beta subunit SucC, class II fumarate hydratase FumC, isocitrate lyase AceA (ABUW_RS14030), type I NADH dehydrogenase subunits NuoC, NdhC, and NuoG (involved in shuttling electrons from NADH to quinones in the electron transport chain [ETC]), F_1_F_o_ ATP synthase subunits AtpA (ABUW_RS18175) and AtpG (**Figs 1E and 2A**). In addition, three proteins responsible for energy-mediated uptake of iron complexes, acinetobactin export ABC transporter permease/ATP-binding subunit BarB, energy transducer TonB (ABUW_RS16655) and ExbB, were all mildly upregulated (log_2_FC values of 1.53, 1.01 and 1.80, respectively; **Figs 1B and 2B**). Conversely, there was a dramatic downregulation in acinetobactin biosynthetic enzymes EntE, BasB, BasF, and utilization protein BauF (log_2_FC values of -5.11, -5.19, -4.61 and -6.13, respectively; **Figs 1B and 2B**).

Stringent response regulators are critical in bacterial physiological activities promoting survival during stress [28], and several were significantly perturbed in response to polymyxin B alone, such as guanosine tetra- or penta-phosphate (p)ppGpp synthetase/hydrolase and RNA polymerase-binding protein DksA. There was a unique upregulation in bifunctional (p)ppGpp synthetase/guanosine-3’,5’-bis(diphosphate) 3’-pyrophosphohydrolase SpoT (ABUW_RS01520; log_2_FC, 2.46) and (p)ppGpp synthetase RelA (ABUW_RS16040; log_2_FC, 1.91) (**Fig 1A**); while, intriguingly, a dramatic downregulation of DksA (log_2_FC, -6.09) (**Fig 1A**).

#### Tripartite condition perturbed energy, stringent response, redox and metal homeostasis in *A. baumannii*

To determine whether THP-1-dMs affected the adaptive responses of interacting bacteria towards subsequent polymyxin B treatment, we compared the bacterial proteome signatures from the tripartite condition (i.e., AB + THP-1-dMs + PMB) with those of the bipartite condition (i.e., AB + PMB). Despite a high level of similarity in DEPs between the two groups, polymyxin-induced upregulation in energy production and ATP-dependent systems observed in the AB + PMB group was abrogated in the AB + THP-1-dMs + PMB group. For example, upregulation in SucC, NuoG, AtpA and AtpG observed in response to polymyxin B alone was reduced to a level approximating that of the untreated control in the AB + THP-1-dMs + PMB group (**Fig 1E**). Notably, the expression of NdhC changed from a log_2_FC of 1.35 when exposed only to polymyxin B to a log_2_ FC of -1.32 in the tripartite condition (**Fig 1E**). Energy-dependent systems that were uniquely upregulated only in response to polymyxin B were also abrogated in the presence of THP-1-dMs. These included multidrug efflux pump membrane proteins (MacA, AdeC, and EmrA/EmrK family multidrug efflux transporter periplasmic adaptor subunit [EmrA, ABUW_RS14655]), ATP-binding protein MlaF (ABUW_RS01880), methionine ABC transporter ATP-binding protein MetN (ABUW_RS07465), lipoprotein-releasing ABC transporter ATP-binding protein LolD, ATP-dependent Clp protease ATP-binding subunit ClpX, energy transducer TonB, and acinetobactin export associated BarB (**Fig 1B, 1C and S1–S3 Tables**).

Interacting bacteria isolated from the AB + THP-1-dMs + PMB group exhibited insignificant differences in KatE, KatG, GrxC, Csp1 and putative cold shock protein (ABUW_RS15360) compared to the untreated control (**Fig 1A**). However, unique proteome remodeling in the iron acquisition systems of interacting bacteria was also observed in this group, as shown by the tripartite-specific upregulation in TonB-dependent siderophore receptor (ABUW_RS18465) and downregulation in siderophore achromobactin biosynthesis protein AcsC (ABUW_RS10625) (**S3 Table**). In the interacting bacteria, the tripartite condition also severely downregulated the copper resistance system multicopper oxidase (ABUW_RS16135; log_2_FC, -3.00) and abrogated infection-specific upregulation in the heavy-metal associated domain-containing protein CopZ (ABUW_RS13140) observed in the AB + THP-1-dMs group (**S1–S3 Tables**). Interestingly, nickel and cobalt homeostasis associated RcnB family protein (ABUW_RS02965) was upregulated (log_2_FC, 3.16) solely in the tripartite condition (**S3 Table**). Of note, upregulation in SpoT and RelA observed in the AB + PMB group was both reduced to a level approximating that of the untreated control in the AB + THP-1-dMs + PMB group (**Fig 1A**).

### Proteomic profiling of THP-1-dMs

We further examined the proteomics of THP-1-dMs samples to better understand the effects of *A*. *baumannii* infection, polymyxin B treatment, and their combination from the host (macrophages) perspective (**S1 Fig**). In total, approximately 4,320 mammalian proteins were identified across all samples involving THP-1-dMs. The PCA score plot revealed that the most distinct separation was between the AB + THP-1-dMs group and the untreated THP-1-dMs control (**S2C Fig**). Expression data of THP-1-dMs in the AB + THP-1-dMs + PMB group was closely clustered with those of the AB + THP-1-dMs group; whereas the data of the THP-1-dMs + PMB group were closely clustered with the untreated THP-1-dMs control (**S2C Fig**). Compared with the untreated control group, there were 98, 94 and 2 DEPs with the AB + THP-1-dMs, AB + THP-1-dMs + PMB, and THP-1-dMs + PMB groups, respectively (**S2D Fig**). Collectively, our data show that the THP-1-dMs proteome was predominantly modulated by AB5075 infection, and 1-h exposure to 30 mg/L polymyxin B had little effect on THP-1-dMs whether alone or in the presence of AB5075 infection. Pathway analysis revealed that infected THP-1-dMs from the AB + THP-1-dMs group were enriched in platelet activation and signaling, high density lipoprotein (HDL) remodeling, heme homeostasis and apoptosis (**S3 Fig**).

**Fig 3:**
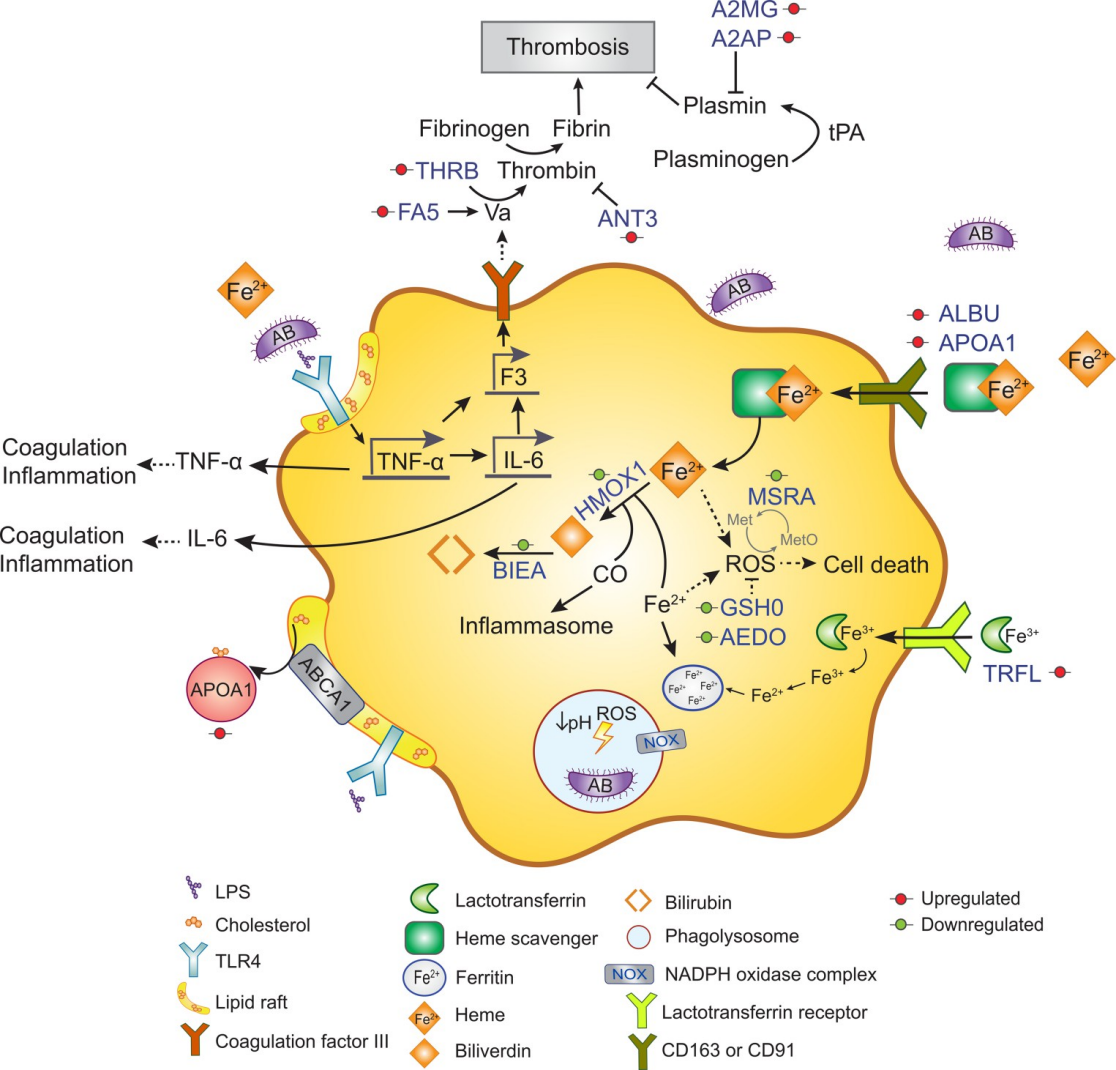
*A*. *baumannii* induces the activation of coagulation cascade in infected THP-1-dMs. Schematic diagram illustrating protein networks involved with the coagulation cascade and iron-heme homeostasis in THP-1-dMs infected by AB5075. Red and green circles indicate upregulated and downregulated differentially expressed proteins, respectively. Data are presented as mean of three biological replicates per experimental group.

Compared to the untreated control, THP-1-dMs in the AB + THP-1-dMs group at 4 h post infection exhibited a significant upregulation of iron-binding proteins, including lactotransferrin (TRFL; log_2_FC, 2.01), heme scavenging associated proteins (e.g., serum albumin [ALBU; log_2_FC, 2.01], hemoglobin subunit alpha [HBA1; log_2_FC, 1.13), and apolipoprotein A-I [APOA1; log_2_FC, 1.74]) (**Fig 3**). However, downregulation was observed in heme catabolic enzymes, such as heme oxygenase 1 (HMOX1; log_2_FC, -1.64) and biliverdin reductase A (BIEA; log_2_FC, -2.68) (**Fig 3**). Notably, host cellular antioxidants were depleted in AB5075-infected THP-1-dMs with downregulation in the modifier subunit of glutamate-cysteine ligase GSH0 (log_2_FC, -1.51) and 2-aminoethanethiol dioxygenase AEDO (log_2_FC, -2.73) (**Fig 3**). Intriguingly, our findings reveal AB5075-induced upregulation of clotting cascade enzymes in infected THP-1-dMs. This was shown by the increased expression of coagulation factor V (FA5; log_2_FC, 3.37), prothrombin (THRB; log_2_FC, 1.27), fibrinolysis inhibitor alpha-2-antiplasmin (A2AP; log_2_FC, 1.45), and alpha-2-macroglobulin (A2MG; log_2_FC, 1.35) in the AB + THP-1-dMs group (**Fig 3**). Of note, anti-coagulant antithrombin-III (ANT3) was also upregulated in infected THP-1-dMs (log_2_FC, 1.52; **Fig 3**).

In the THP-1-dMs + PMB group, THP-1-dMs downregulated transcriptional regulator SWI/SNF-related matrix-associated actin-dependent regulator of chromatin subfamily E member 1 (SMCE1; log_2_FC, -2.08) and upregulated intracellular trafficking associated TBC1 domain family member 15 (TBC15; log_2_FC, 1.83). When the AB + THP-1-dMs + PMB and AB + THP-1-dMs groups were compared, downregulation was revealed in glycolytic enzyme ATP-dependent 6-phosphofructokinase, muscle type (PFKAM; log_2_FC, -4.23), while upregulation in phospholipid phosphatase 6 (PLPP6; log_2_FC, 2.56).

### Impaired bacterial oxidative stress resistance, iron homeostasis and stringent response regulation enhanced polymyxin B killing and reduced bacterial virulence *in vivo*

**Fig 4:**
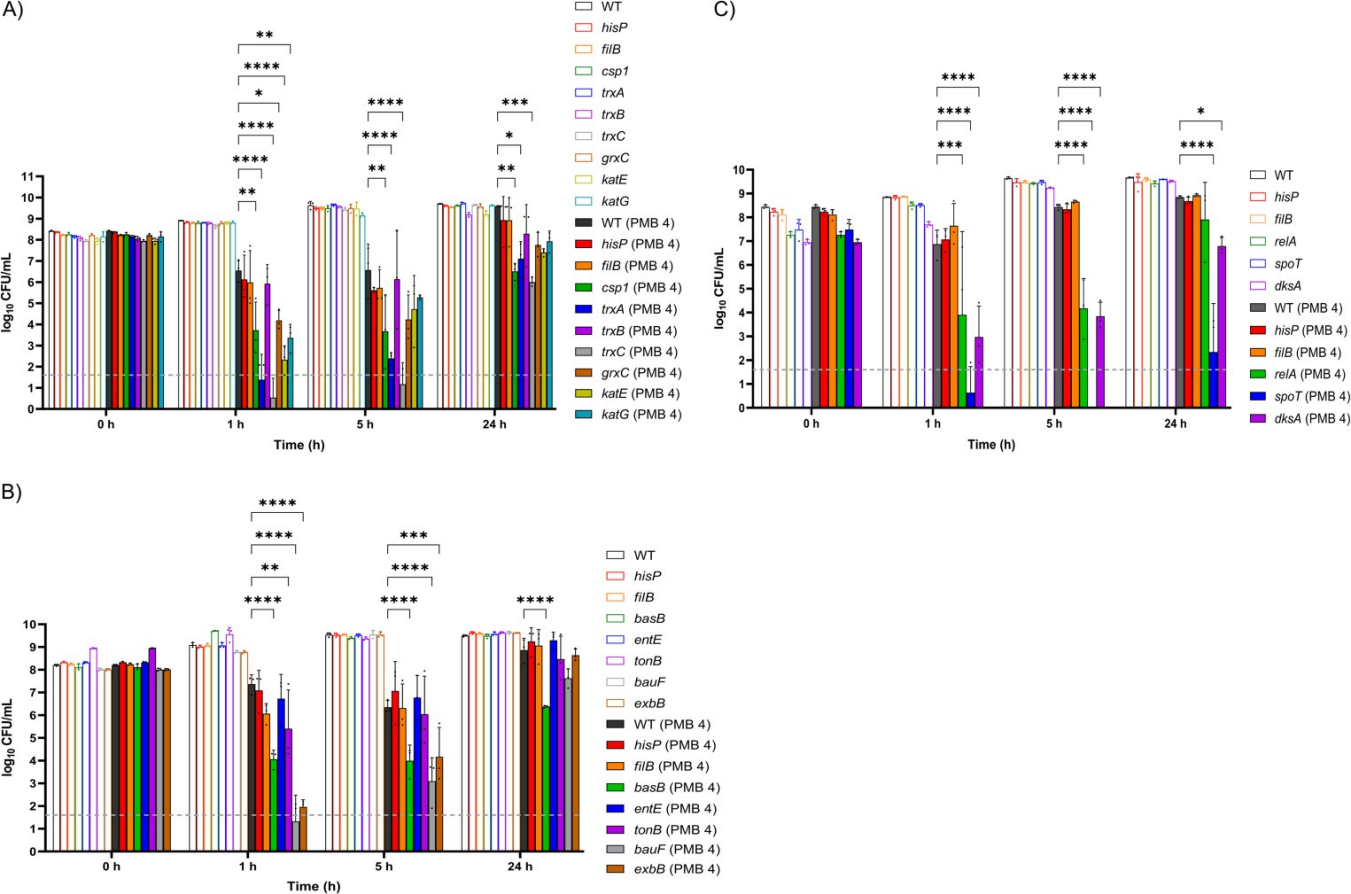
Impaired stress response machineries in *A*. *baumannii* enhances polymyxin killing. Time-kill profiles of AB5075 transposon mutants with disrupted **A**) redox stress resistance genes; **B**) iron acquisition genes; **C**) stringent response regulatory genes following 4 mg/L polymyxin B (PMB) treatment. Data shown as mean ± SD of three biological replicates. Two-way ANOVA with Tukey’s HSD post-hoc test, * p <0.05, ** p <0.01, *** p <0.001, **** p <0.0001. Dashed line indicates the limit of detection.

Next, we investigated the role of identified candidate targets in affecting bacterial killing by polymyxin B. As the wild-type *A*. *baumannii* AB5075 exhibited rapid tolerance towards polymyxins [29], 4 mg/L polymyxin B (i.e., 16× minimum inhibitory concentration [MIC]) was employed to compare the *in vitro* killing kinetics of the wild-type and mutants. Consistent with our hypothesis generated from the proteomics results above, enhanced bacterial killing by polymyxin B was observed with the selected AB5075 transposon mutants in redox stress resistance, namely those with disrupted *trxC*, *trxA*, *katE*, *katG* and *csp1* (**Fig 4A**). At 1 and 5 h post polymyxin B treatment (4 mg/L), *trxC* and *trxA* mutants both showed 4- to 6 log_10_ CFU/mL additional killing compared to that of the wild-type, with >3 log_10_ CFU/mL additional killing at 24 h (**Fig 4A**). Enhanced polymyxin B killing was also observed with the *katG* and *katE* mutants at 1 h (at least 3 and 4 log_10_ CFU/mL), the *csp1* mutant at 1, 5 and 24 h (at least ~3 log_10_ CFU/mL), and the *grxC* mutant at 1 and 5 h (>2 log_10_ CFU/mL) (**Fig 4A**). Polymyxin B time-kill kinetics of AB5075 mutants with T26 transposon-mutated pseudogenes *hisP* (ABUW_RS06545) or *filB* (ABUW_RS15750) mirrored those of the wild-type strain (**Fig 4**).

AB5075 transposon mutants with disrupted iron acquisition genes were more susceptible to the initial killing by polymyxin B, compared to that of the wild-type strain. Much better polymyxin B killing was evident against *bauF* and *exbB* mutants, such as at 1 h with additional killing of 6 and 5 log_10_ CFU/mL, respectively (**Fig 4B**). With the *basB* mutant, >2 log_10_ CFU/mL killing was observed at 1 and 5 h, and 1.95 log_10_ CFU/mL killing with the *tonB* mutant at 1 h (**Fig 4B**).

**Fig 5:**
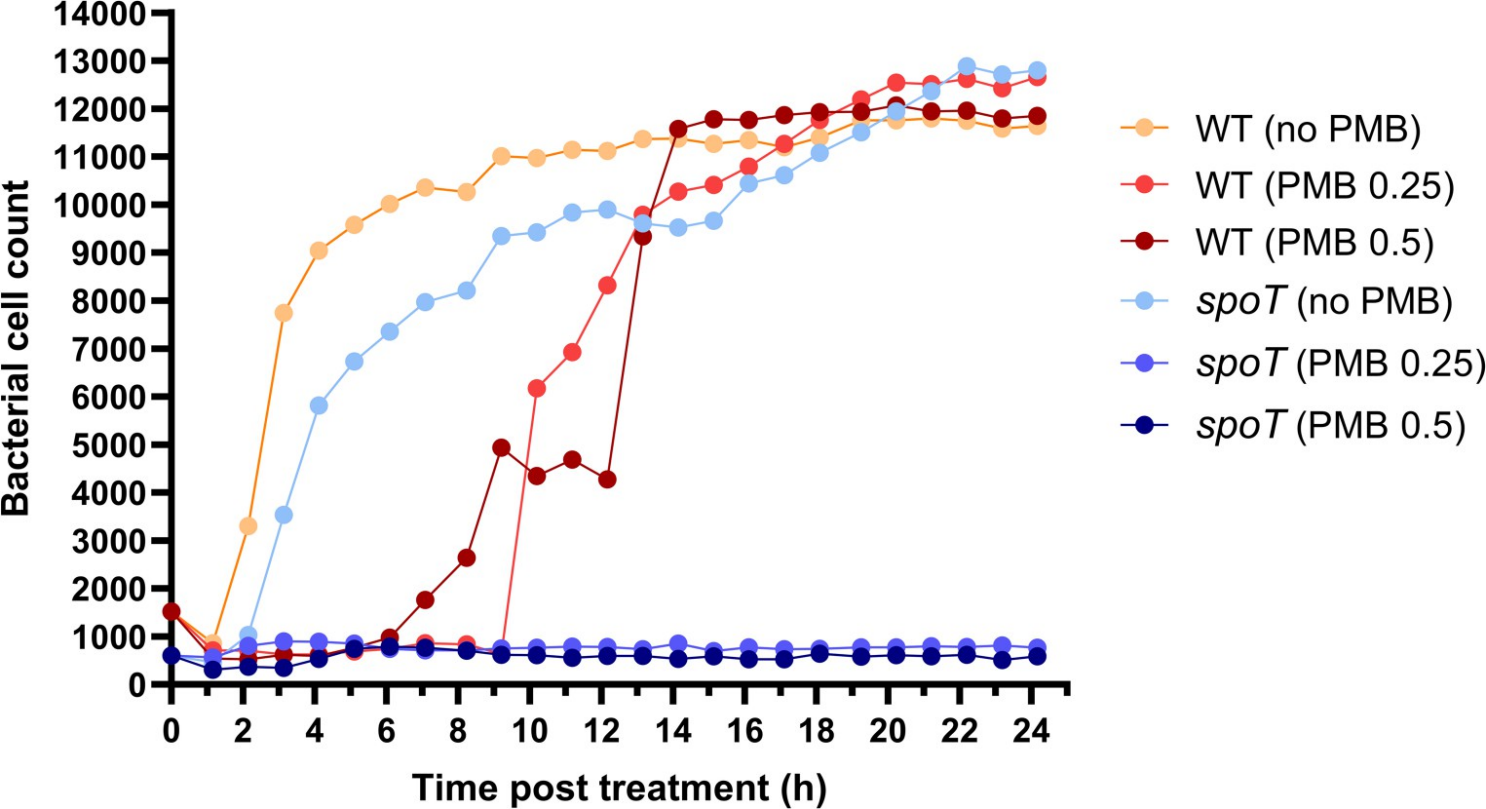
Time-lapse live-cell imaging reveals different growth kinetics of *spoT* mutant following polymyxin treatment. ImageJ was employed for imaging analysis and cell count of AB5075 wild-type (WT) and *spoT* mutant following polymyxin B (PMB) treatment at 0.25 or 0.5 mg/L, and no treatment (i.e., controls).

Notably, with polymyxin B treatment alone, inactivation in the bifunctional (p)ppGpp synthetase/hydrolase *spoT* resulted in the greatest enhancement of killing (6–8 log_10_ CFU/mL) in the tested mutants across all timepoints (**Fig 4C**). This dramatically enhanced killing matched the observations of our time-lapse imaging study which showed low cell counts of the *spoT* mutant across 24 h when treated with polymyxin B at 0.25 and 0.5 mg/L (**Fig 5**). In contrast, at both polymyxin B concentrations growth of the wild-type AB5075 was suppressed only over the first 7–10 h followed by rapid regrowth that approximated the control values by ~14 h (**Fig 5**). Interestingly, our imaging data revealed that, compared to the wild-type AB5075, *spoT* mutants exhibited a relatively smaller cell size at 0 h. AB5075 wild-type showed a mean (± SD) cell area of 3.35 ± 0.92 μm^2^ at 0 h; whereas *spoT* mutants exhibited a much lower mean (± SD) cell area of 2.58 ± 1.13 μm^2^ (Welch’s *t*-test, p <0.0001). At 24 h, the mean (± SD) cell area of *spoT* mutants treated with 0.5 mg/L polymyxin B did not increase (2.98 ± 1.43 μm^2^); while the mean (± SD) cell area of untreated *spoT* mutants increased to 4.91 ± 3.42 μm^2^, comparable to those of wild-type cells regardless of polymyxin B treatment (4.31 ± 2.56 μm^2^, 4.56 ± 2.81 μm^2^ and 4.58 ± 2.85 μm^2^ for untreated, 0.25 mg/L and 0.5 mg/L polymyxin B treated wild-type, respectively).

**Fig 6:**
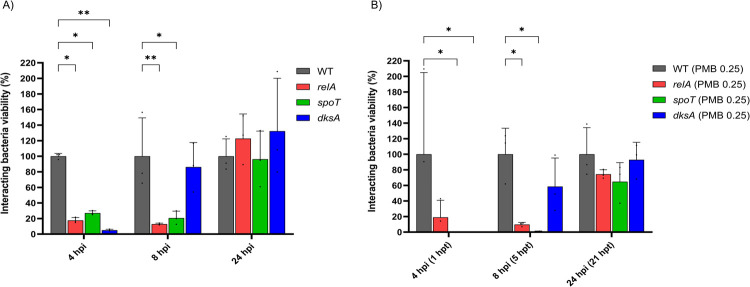
Impaired stringent response reduces interacting bacterial growth in the presence of THP-1-dMs. Interacting bacterial growth of AB5075 wild-type (WT) and transposon mutants with disrupted stringent response regulatory genes following MOI 1,000 infection of THP-1-dMs in the **A**) absence and **B**) presence of 0.25 mg/L polymyxin B (PMB) treatment. Data shown as mean ± SD of three biological replicates compared to that of the wild-type. Two-way ANOVA with Tukey’s HSD post-hoc test, * p <0.05, ** p <0.01.

Inactivation of stringent response regulator *dksA* also led to enhanced polymyxin B killing, with ~4 log_10_ CFU/mL additional killing of the *dksA* mutants at 1 and 5 h and 2 log_10_ CFU/mL additional killing at 24 h (**Fig 4C**). Additionally, *relA* mutants exhibited reduced tolerance to initial polymyxin B exposure with additional killing of 2.97 and 4 log_10_ CFU/mL at 1 and 5 h, respectively (**Fig 4C**). Subsequently, we compared the antibacterial activity of polymyxin B against the three stringent response-associated mutants *spoT*, *dksA*, and *relA* to that of the wild-type in a macrophage infection microenvironment. Herein, 0.25 mg/L polymyxin B (i.e., 1×MIC) was employed to prevent overkill of interacting bacterial cells and allow comparison of the antibiotic killing dynamics between the interacting wild-type and mutants. Interestingly, during early THP-1-dMs infection in the absence of polymyxin B, all three mutants had significantly fewer interacting bacteria at 4 h post infection, compared to that of the wild-type (**Fig 6A**). At 4 h (i.e., following 1 h of polymyxin B treatment), the antibiotic treated wild-type group exhibited 2.7 log_10_ CFU/mL interacting bacteria; however, at this time no interacting bacteria were detected with the *dksA* mutant while the *spoT* mutant was reduced by 99.5% compared to that of the wild-type (**Fig 6B**). At 8 h (i.e., 5 h post polymyxin B treatment), the viability of the interacting *spoT* and *relA* mutants was significantly reduced (by >98% and 90%, respectively), compared to that of the wild-type (**Fig 6B**).

**Fig 7:**
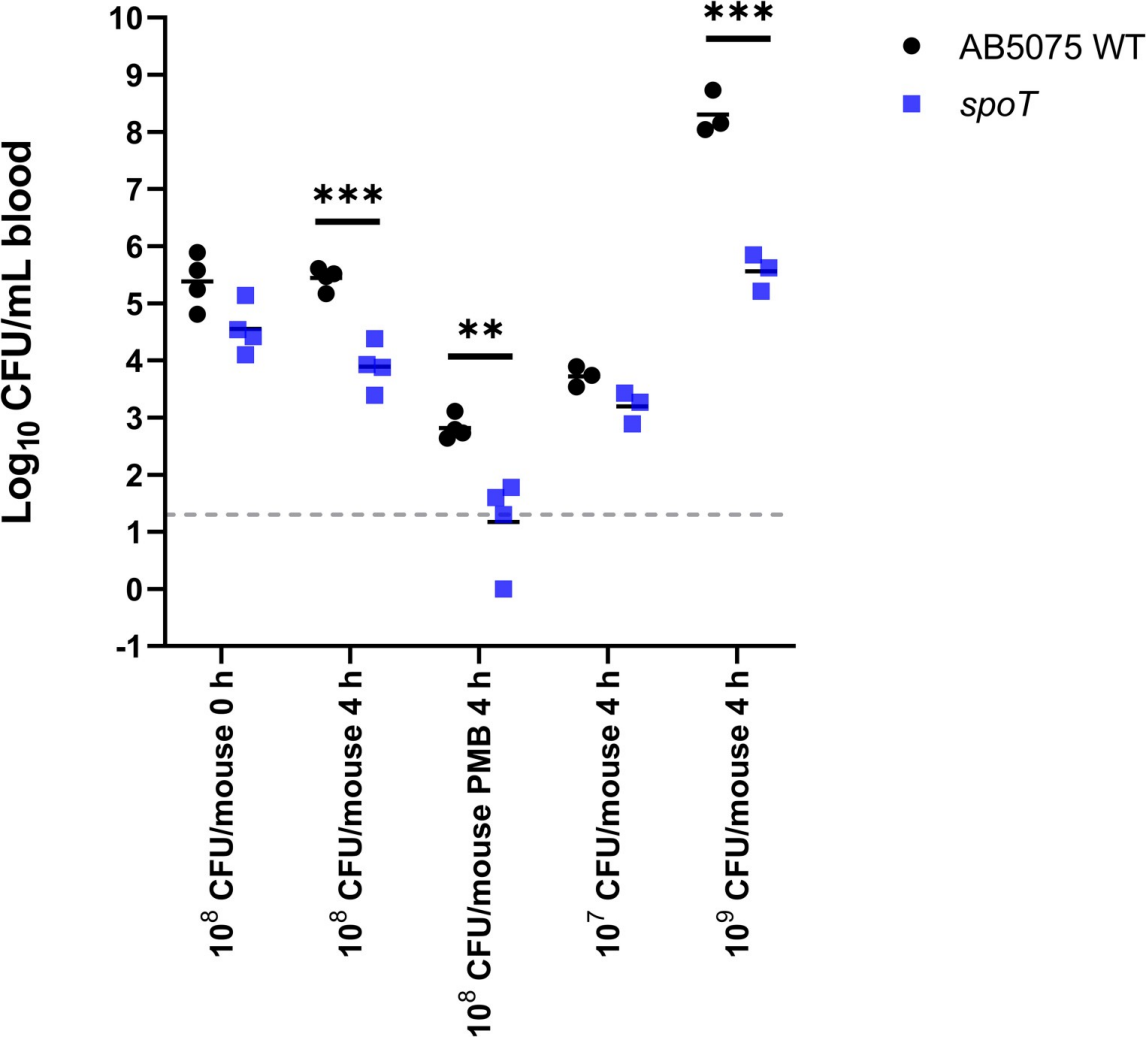
*spoT* mutant exhibits reduced virulence *in vivo*. Bacterial load of AB5075 wild-type (WT) and *spoT* mutant in blood in a mouse bacteremia model with and without polymyxin B (PMB) treatment (4 mg/kg, intravenous). At least three biological replicates per experimental group were examined. Multiple unpaired *t*-tests, ** p <0.01, *** p <0.001. Dashed line indicates the limit of detection.

To validate our *in vitro* findings, we employed *spoT* mutants as an example to examine the role of bacterial stringent response in the survivability in an immunocompetent mouse bacteremia model. At 6 h after bacterial inoculation (in the absence of polymyxin B), significantly reduced bacterial load in blood was observed in mice infected by the *spoT* mutant at both 10^8^ and 10^9^ CFU/mouse inoculum, compared to that of AB5075 wild-type infection control (**Fig 7**). Using the same mouse bacteremia model with intravenous polymyxin B (4 mg/kg, the maximum dose in mice via intravenous administration), the average bacterial count of *spoT* mutant in blood at 4 h reduced to below the limit of detection of 1.30 log_10_ CFU/mL, showing at least >1.65 log_10_ CFU/mL reduction compared to that of the wild-type AB5075 (**Fig 7**).

## Discussion

Our study is the first to examine the complex tripartite interplay among *A*. *baumannii*, macrophages and an antibiotic using an *in vitro* host-pathogen-drug model. Using correlative proteomics, we discovered several potential pathogen-directed or host-directed candidate proteins and pathways for future therapeutic targeting. Correlative proteomic profiling of infected macrophages and their interacting bacteria allowed an integrative analysis of the interaction between innate immune cells and pathogen during the infection. A high multiplicity of infection (MOI; 1,000) was employed to achieve a sufficient number of interacting bacterial cells for proteomic analysis. Considering the rapid killing by polymyxins [30], 4 h post infection and 1 h post treatment was examined. The polymyxin concentration of 30 mg/L used in this proteomic study is achievable in lung epithelial lining fluid in patients following aerosolized polymyxins [31]. We identified diverse responses and tolerance strategies of *A*. *baumannii* towards polymyxin B, macrophages, and the combination. It was revealed that macrophages and polymyxin B exhibited complementary effects to disarm several tolerance and survival strategies of *A*. *baumannii*, most notably oxidative stress resistance, copper tolerance, bacterial iron acquisition and stringent response regulation systems. Importantly, we have identified several novel pathogen-directed targets from the redox stress response system, iron acquisition and stringent response regulation that warrant further investigations for developing potential therapeutic strategies to enhance polymyxin efficacy against MDR *A*. *baumannii*.

A major finding here is the association of polymyxin activity with (p)ppGpp-mediated stringent response in AB5075. As nutritional stress (e.g., starvation of amino acids, carbon, fatty acids, phosphate and iron) can trigger (p)ppGpp biosynthesis [28,32–34], it is very likely that the upregulation of bifunctional (p)ppGpp synthetase/guanosine-3’,5’-bis(diphosphate) 3’-pyrophosphohydrolases SpoT and (p)ppGpp synthetase RelA in *A*. *baumannii* after polymyxin treatment (**Fig 1A**) was triggered by polymyxin-induced nutritional stress. This is supported by the downregulation of proteins involved in amino acid biosynthesis, glycolysis, and iron uptake systems (**Fig 1B, 1E and 1F**), as well as the previously reported transcriptional perturbations of fatty acids biosynthesis in *A*. *baumannii* following polymyxin treatment [35]. Alarmone (p)ppGpp promotes bacterial survival in adverse growth conditions via global reprogramming of bacterial cellular and metabolic activities, during which (p)ppGpp binds to the RNA polymerase to regulate gene expression [36]. The outcome of this process is reduced transcription of ribosomal and transfer RNAs necessary for translation in order to balance protein biosynthesis with nutrient availability [36–38]. In the present study, we observed the downregulation of translational machinery such as elongation factors FusA, EfP, and Tsf (ABUW_RS06040) in polymyxin-treated *A*. *baumannii*, representative of stringent response activation (**Fig 1G**). The crucial role of (p)ppGpp-mediated stringent response in governing oxidative stress resistance, heat shock response, antibiotic tolerance and virulence has been recently reported in *F*. *tularensis*, *B*. *subtilis* and *P*. *aeruginosa* [39–41]. Several recent studies on *relA*-deleted *A*. *baumannii* unveiled a global regulatory role of *relA* in bacterial adherence, biofilm formation, and Ade multidrug efflux family; as well as a reduced virulence in a *Galleria mellonella* model and neutropenic murine pneumonia model [42–44]. Here, we firstly identified the key role of (p)ppGpp-mediated stringent response in the host-pathogen-drug interplay using proteomics, and subsequently demonstrated that the transposon mutants *relA* (ABUW_RS16040) and especially *spoT* (ABUW_RS01520) in AB5075 were much more susceptible to polymyxin killing (**Fig 4C**). Our findings highlight the importance of the (p)ppGpp-mediated stringent response in polymyxin tolerance in *A*. *baumannii*. The prolonged (up to 24 h) suppression of bacterial replication of polymyxin-treated *spoT* mutants observed in our time-lapse imaging study (**Fig 5**) further highlights the crucial role of *spoT* mediated nutritional regulation and downstream signaling in polymyxin tolerance.

Besides (p)ppGpp synthetase/hydrolase, our study also highlights the critical role of another stringent response regulator DksA in mediating *A*. *baumannii* tolerance towards polymyxins. DksA exerts a synergistic regulatory role in the (p)ppGpp-mediated stringent response in *Salmonella enterica* [45]. In nutritionally-starved *Salmonella*, DksA plays a key role in regulating the NAD(P)H/NAD(P)^+^ redox balance that fuels downstream antioxidant systems, including the thioredoxin superfamily, through fine-tuning discrete steps in central carbon metabolism [46]. In line with this notion, we discovered dramatic downregulation of DksA protein abundance in polymyxin-treated *A*. *baumannii* along with the downregulation of NAD(P)H-dependent antioxidant systems, including thioredoxin and glutaredoxin, and protein repair associated Dsb systems (**Figs 1A and 2A**). Notably, our transposon mutant study further confirmed that singly-gene disruption of *relA*, *spoT* or *dksA* significantly decreased the growth of interacting *A*. *baumannii* during early macrophage infection. Consistent with our tripartite proteomics data showing complementary action between macrophages and polymyxins in disarming the upregulation of (p)ppGpp synthetase/hydrolase SpoT (**Fig 1A**), a low concentration of polymyxin B (0.25 mg/L) in the THP-1-dMs infection system is sufficient to significantly reduce the survival of interacting *spoT* mutants compared to the wild-type strain (**Fig 6B**). Importantly, our *in vivo* study further validated that disruption of *spoT* reduced *A*. *baumannii* survivability and polymyxin treatment cleared most of the *spoT* mutants in blood of the infected mice (**Fig 7**). Collectively, these results show a potential therapeutic benefit of targeting the *A*. *baumannii* stringent response regulation (not limited to *relA*) to reduce bacterial virulence and enhance polymyxin killing, including macrophage-associated interacting bacteria.

Furthermore, our study also discovered several proteins of oxidative stress resistance and metal homeostasis systems that underlie *A*. *baumannii* pathogenesis and polymyxin tolerance. During infection, *A*. *baumannii* can induce reactive oxygen species (ROS) production in macrophages [47] and phagocytes such as macrophages employ respiratory burst (i.e., a rapid increase in the production of ROS during phagocytosis) to kill bacteria [48]. Our results here highlight the distinct oxidative stress resistance machineries in *A*. *baumannii* in response to macrophages and polymyxins. Interacting *A*. *baumannii* may utilize cold shock proteins and proteins of the thioredoxin superfamily to facilitate their tolerance against redox stress and lysosomal pH encountered in macrophages (**Fig 1A**). This finding is new as the use of cold shock proteins to resist redox stress has not been reported in *A*. *baumannii*. In the literature, cold shock protein CspA in *Brucella melitensis* plays an important role in resistance to acidic and hydrogen peroxide stresses, allowing bacterial replication in J774.A1 murine macrophages [49]. The thioredoxin superfamily includes both the thioredoxin and glutathione/glutaredoxin systems, and serves as a key antioxidant system through regulation of protein dithiol/disulfide balance [50,51]. It is also involved in DNA and protein repair by transferring reducing equivalents to ribonucleotide reductase, Msr and Dsb systems, as well as regulating the activity of redox-sensitive transcription factors [52]. Our finding of the upregulation of TrxA in interacting *A*. *baumannii* with macrophages is in agreement with the known roles of TrxA in facilitating bacterial intracellular replication in epithelial cells or macrophage-like cells, resistance to hydrogen peroxide, and *in vivo* virulence of several bacterial species, including *A*. *baumannii* [53–55]. Moreover, we demonstrated here a critical link between *A*. *baumannii* oxidative stress resistance systems and polymyxin antibacterial efficacy. In *A*. *baumannii* polymyxins induce upregulation of H_2_O_2_-hydrolyzing catalases and their killing is potentially mediated by a hydroxyl radical death pathway [56,57]. Enhanced bacterial killing of *A*. *baumannii* has been reported with a combination of colistin and pro-oxidant curcumin that synergistically increased ROS production [58]. The results of our transposon mutant time-kill kinetics highlight the crucial role of catalases (KatE, KatG), thioredoxin (TrxA, TrxC) and glutaredoxin (GrxC) in *A*. *baumannii* to resist polymyxins (**Fig 4A**). Notably, the increased expression of catalases, thioredoxin superfamily, and cold shock proteins in interacting *A*. *baumannii* was abrogated in the tripartite condition (**Fig 1A**), indicating complementary antibacterial activity between macrophages and polymyxins. Together, our findings highlight the potential of targeting such oxidative stress resistance systems as to enhance polymyxin activity.

Maintenance of metal homeostasis is crucial for both the host and pathogen [59]. Metals serve as cofactors in diverse biochemical processes, but are toxic at high concentrations [59,60]. During bacterial infection, the host innate immune system could harness copper toxicity as an antimicrobial strategy [61]. The mechanisms underlying copper toxicity include rapid inactivation of iron-sulfur dehydratase family [62]; incorrect periplasmic disulfide bond formation [63]; and Fenton and Haber-Weiss reaction-mediated hydroxyl radical generation [64]. Attenuated virulence of *A*. *baumannii* mutants with disrupted copper resistance genes in *Galleria mellonella* and mouse pneumonia infection models highlight the importance of copper tolerance in bacterial virulence within host [65,66]. Our proteomics results indicate that interacting *A*. *baumannii* cells likely utilized glutaredoxins (GrxC, GrxD) and a putative copper chaperone CopZ (ABUW_RS13140) as a copper tolerance strategy to survive within macrophages. Glutaredoxin minimizes the incorrect formation of disulfide bonds in cellular proteins resulting from copper-induced oxidation of sulfhydryl groups [67,68]. In *Escherichia coli*, inactivation of the disulfide bond isomerase *dsbC* increases the sensitivity to copper toxicity through impairing bacterial ability to resolve copper-catalyzed non-native disulfides [63]. Interestingly, our proteomics results indicate that polymyxin treatment likely sensitizes interacting bacteria towards host-induced copper toxicity by preventing the upregulation of putative copper chaperone CopZ, and by inducing downregulation of the bacterial Dsb system (**S1–S3 Tables and Fig 1A**). Downregulation of multicopper oxidase (ABUW_RS16135) was only observed in the tripartite condition (**S3 Table**). ABUW_RS16135 encodes for a CopA homolog (87.4% identity with CopA [AMQ95338.1] in *A*. *baumannii*), and shares similarity with periplasmic copper oxidase PcoA (BCZ13020.1) in *A*. *baumannii* (83.6% identity) which detoxifies oxidizing Cu^1+^ to less damaging Cu^2+^ ions [69,70]. Such tripartite-specific downregulation in CopA (ABUW_RS16135) suggests a novel cooperative action of macrophages and polymyxins in perturbing *A*. *baumannii* defense against toxic cuprous ions. Overall, our findings indicate a complementary antibacterial mechanism between macrophages and polymyxins against *A*. *baumannii* by both impairing bacterial copper detoxification.

In addition, mammalian hosts can activate nutritional immunity through restricting the availability of nutrient metals to invading bacteria [71]. Our proteomics results indicate that infected macrophages induced host iron sequestration via increased cellular uptake of lactotransferrin and heme scavengers from the environment (**Fig 3**), corroborating with the observed upregulation of lactotransferrin and heme scavenging associated haptoglobin in an *A*. *baumannii* lung infection proteomic study in mice [72]. Attenuation of bacterial siderophore biosynthesis, supplementation of iron competitors (i.e., gallium) and iron scavenger (i.e., host-derived transferrin) can reduce bacterial survival and virulence (e.g., *A*. *baumannii*) *in vitro* and *in vivo* [73–75]. Additionally, our host proteomics results revealed that *A*. *baumannii*-infected macrophages downregulated the host heme catabolism, which possibly activated innate immune responses [76]. Depletion of heme catabolic enzymes HMOX1 and BIEA in infected macrophages leads to intracellular accumulation of pro-oxidative and pro-inflammatory free labile heme [77,78]. Free heme may act as damage-associated molecular patterns (DAMPs), amplifying the innate immune response through induction of toll-like receptor (TLR)-mediated production of pro-inflammatory tumor necrosis factor TNF-α, and NADPH oxidase (NOX)-dependent ROS production in phagocytes [76,79]. In line with this, the recent proteomic study of *A*. *baumannii* lung infection in mice indicated the activation of NOX signaling as an important host defense strategy; whereas the role of heme in this process warrants further investigation [72,80]. On the other hand, inefficient neutralization of excessive free heme leads to heme toxicity and sepsis [81]. In addition, an overwhelming pro-inflammatory response during *Acinetobacter* infection may activate the coagulation cascade and lead to thrombosis and disseminated intravascular coagulation [82]. Our proteomics findings indicate that better understanding of *A*. *baumannii*-induced coagulation may assist with the treatment of *A*. *baumannii* infection.

In the present study, polymyxin-treated *A*. *baumannii* cells modulated their ferric iron acquisition systems by downregulating acinetobactin biosynthetic enzymes at 1 h post treatment (**Figs 1B and 2B**), indicating bacterial attempt to reduce the iron-catalyzed Fenton reaction and ROS production during early polymyxin exposure. Similarly, downregulation in siderophore-mediated iron acquisition in interacting bacterial cells (**Figs 1B and 2B**) and extracellular bacterial cells [83] following macrophage infection indicates a common stress tolerance strategy in *A*. *baumannii* through regulating the siderophore activities. Iron is an essential enzymatic cofactor in a wide variety of fundamental metabolic pathways, including antioxidant systems (e.g., iron-containing superoxide dismutases and catalases), central carbon metabolism (e.g., iron-sulfur domain-containing aconitase, succinate dehydrogenase, and NADH dehydrogenase), and DNA biosynthesis and repair [84–86]. Therefore, suppression of iron uptake can cause iron starvation and subsequent activation of bacterial iron acquisition system as a feedback mechanism. In agreement with this, we observed that polymyxin-treated *A*. *baumannii* upregulated their TonB protein and MotA/TolQ/ExbB proton channel for active uptake of ferric iron complexes, likely to cope with iron starvation (**Figs 1B and 2B**). Further, our AB5075 transposon mutant screening demonstrated, for the first time, the importance of iron acquisition proteins BasB, EntE, MotA/TolQ/ExbB proton channel (ExbB) and iron assimilation protein BauF in mediating polymyxin tolerance in AB5075. Indeed, disruption of bacterial heavy metal homeostasis by ionophore 2-(dimethylamino) methyl-5,7-dichloro-8-hydroxyquinoline (PBT2) dramatically sensitizes polymyxin-resistant Gram-negative (e.g., *A*. *baumannii*) to polymyxin killing [87]. Taken together, our findings highlight the therapeutic potential of targeting heavy metal homeostasis in *A*. *baumannii* (e.g., iron acquisition) to enhance polymyxin killing.

## Conclusions

With the emergence of polymyxin-resistant *A*. *baumannii* threatening the utility of this important last-line class of antibiotics, innovative strategies to preserve their clinical efficacy (e.g., development of novel adjuvant therapies) are urgently needed. Our proteomics findings in the *A*. *baumannii*-macrophage-polymyxin interactions discovered that key bacterial stress responses and tolerance strategies towards polymyxins and/or macrophages may serve as pathogen-directed therapeutic target candidates. In particular, the stringent response regulator *spoT* is exemplified as how a single gene/protein modulation can affect *A*. *baumannii* tolerance towards polymyxins *in vitro* and in mice. Our findings highlight the significant potential of deciphering the complex tripartite interactions between host, pathogen and drug in optimizing antibiotic use and expediting antibiotic discovery against MDR pathogens.

## Materials and methods

### Bacterial strains, media and antibiotic

*Acinetobacter baumannii* AB5075 (wild-type [WT]) is a recently characterized MDR isolate collected from a wound of a patient at the Walter Reed Army Medical Center [88]. The *A*. *baumannii* AB5075-UW transposon mutant library with targeted gene disruption via single T26 transposon insertion was purchased from the University of Washington [89]. The AB5075 transposon mutants used in this study are shown in **S4 Table** and were validated using colony polymerase chain reaction. Logarithmic-phase cultures of AB5075 WT were prepared from -80°C frozen stock, plated onto nutrient agar (NA), and incubated aerobically at 37°C for 16 to 18 h. A single bacterial colony was then inoculated into cation-adjusted Mueller-Hinton broth (CaMHB) and incubated overnight at 37°C with shaking (200 rpm) for 16–18 h. Overnight cultures were diluted 100-fold in pre-warmed CaMHB and further grown at 37°C to reach an optical density (OD) of 0.5 at 600 nm. AB5075 transposon mutants were similarly prepared using Luria-Bertani (LB) agar supplemented with 10 mg/L tetracycline hydrochloride (Catalog: T7660-5G; Sigma). Polymyxin B sulphate (PMB; Catalog: 86–40302) was obtained from Betapharma (Shanghai, China). Sterile stock solutions were prepared using Milli-Q water (Millipore, USA) filtered through a 0.22-μm syringe filter (Sartorius, Germany).

### Mammalian cell line

THP-1 (ATCC TIB-202), a human leukemia monocytic cell line, was maintained in Roswell Park Memorial Institute (RPMI) 1640 medium supplemented with 25 mM of 4-(2-hydroxyethyl) piperazine-1-ethanesulfonic acid (HEPES) buffer solution and 10% fetal bovine serum (FBS; Lot 15703; Bovogen, Victoria, Australia) and incubated at 37°C in a humidified atmosphere containing 5% CO_2_. THP-1 cells were differentiated into macrophage-like THP-1 cells (THP-1-dMs) via 48 h of treatment with 25 nM of phorbol 12-myristate 13-acetate (PMA; Santa Cruz Biotechnology; Texas, USA), after which the media was removed and replaced with fresh pre-warmed RPMI 1640 media supplemented with 25 mM HEPES and 10% FBS. Differentiated cells were allowed to rest for at least 24 h in PMA-free media prior to experiments.

### THP-1-dMs infection and polymyxin treatment

To obtain sufficient interacting bacterial proteins for mass spectrometric analysis, AB5075 infection of THP-1-dMs was performed in two T175 flasks (Corning; New York, USA) at a multiplicity of infection (MOI) of 1,000; the content of each flask was subsequently pooled to obtain a single sample. For host cell proteomics samples, infection was performed in 6-well plates (Corning; Jiangsu Province, China) and proceeded for 4 h in RPMI 1640 media supplemented with 25 mM HEPES and 10% heat-inactivated FBS at 37°C (5% CO_2_). For antibiotic-treated samples, polymyxin B 30 mg/L was added at 3 h post infection meaning cells were exposed to polymyxin B for 1 h. At 4 h, cell monolayers were washed twice with ice cold Dulbecco’s phosphate-buffered saline (DPBS, 1×; Gibco, Paisley, UK) to remove non-interacting extracellular bacteria. To isolate interacting bacteria from infected THP-1-dMs, cells were firstly lysed in 1% Triton X-100 for 1 h on ice. A two-step differential centrifugation strategy was then performed to separate interacting bacterial cells from host cell debris [90]. Firstly, the sample was centrifuged at 600 × *g* and 4°C for 5 min to pellet host cell debris. Second, the supernatant containing interacting bacteria released from lysed host cells was centrifuged at 3,220 × *g* for 20 min (at 4°C) to pellet bacteria. The resulting bacterial pellet was thrice washed with ice cold DPBS (1×) to minimize contamination from both residual host proteins and Triton X-100. The final bacterial pellets were stored at -20°C until further analysis. The respective controls of THP-1-dMs and AB5075 were treated in the same manner. Three biological replicates were included for each experimental condition.

### LC-MS/MS sample preparation and analysis

To extract proteins from infected THP-1-dMs, cell monolayers were washed twice with ice cold DPBS (1×) to remove residual media prior to the treatment with lysis buffer 1% sodium deoxycholate (SDC; Sigma, D6750-100G) in 100 mM HEPES (pH 8.5). For bacterial protein extraction, the previously isolated bacterial pellet was resuspended with 1% SDC in 100 mM HEPES (pH 8.5). SDC-treated samples were heated at 95°C for 10 min prior to probe sonication at output strength 3 and 50% duty cycle. Sample protein was then quantified using the Pierce BCA Protein Assay Kit (Thermo Fisher Scientific; Illinois, USA). The total protein was normalized to 150 μg followed by protein denaturation and alkylation with 10 mM bond-breaker tris-(2-carboxyethyl) phosphine (TCEP) and 40 mM chloroacetamide (CAA). Samples were then subjected to overnight trypsin digestion at 37°C. SDC was subsequently removed by 1% formic acid precipitation and the sample peptides purified using the Eppendorf PerfectPure C18 ZipTip protocol. Acetonitrile in eluted samples was removed by a vacuum concentrator. Peptide samples were then reconstituted in 0.1% formic acid and sonicated in a water bath prior to liquid chromatography-tandem mass spectrometry (LC-MS/MS) analysis.

Mass spectrometric analysis were performed by the Monash Proteomics & Metabolomics Facility. Briefly, a Dionex UltiMate 3000 RSLCnano system was employed with an Acclaim PepMap RSLC analytical column (75 μm × 50 cm, nanoViper, C18, 2 μm, 100Å; Thermo Scientific) and an Acclaim PepMap 100 trap column (100 μm × 2 cm, nanoViper, C18, 5 μm, 100Å, Thermo Scientific). Tryptic peptides of bacterial cells were analyzed on an Orbitrap Fusion Tribrid Mass Spectrometer (Thermo Fisher Scientific) operated in data-dependent acquisition (DDA). For AB5075, raw files were analyzed with MaxQuant v1.6.5.0 and statistical analysis of the label-free quantification (LFQ) intensities was performed in Perseus v1.6.2.3. The AB5075 proteome was annotated using the NCBI Assembly Database (https://ftp.ncbi.nlm.nih.gov/genomes/all/GCF/000/963/815/GCF_000963815.1_ASM96381v1/). For THP-1-dMs, the tryptic peptides were analyzed on a QExactive Plus Mass Spectrometer (Thermo Scientific) operated in data-independent acquisition (DIA) mode. The raw files of THP-1-dMs data were analyzed with Spectronaut v13 Laika (Biognosys) to obtain relative LFQ values using in-house standard parameters. The expression dataset of each experimental groups was visualized with a principal component analysis (PCA) score plot generated using MetaboAnalyst and Venn diagram generated using *InteractiVenn* [91,92]. Differences in protein expression levels were evaluated through comparison of the mean LFQ intensities among all experimental groups and expressed as the log_2_ fold change (log_2_ FC). Significance was determined using a two-sided, two-sample *t*-test with a false discovery rate (FDR) adjusted p-value. Differentially expressed proteins (DEPs) with a log_2_FC >1 or <-1 and FDR <0.05 were considered statistically significant. Statistically significant bacterial DEPs were subjected to pathway analysis according to Clusters of Orthologous Groups (COGs) and predicted by eggNOG [93] and Kyoto Encyclopedia of Genes and Genomes (KEGG) pathways using R. Statistical significance of enrichment analysis was examined using Fisher’s Exact Test (FDR <0.2). THP-1-dMs DEPs were functionally analyzed on KEGG and Reactome pathways using WebGestalt with the pathway enrichment significance set at FDR <0.05 using the Benjamini-Hochberg method [94,95].

### Functional investigation of AB5075 mutants

The time-kill kinetics of polymyxin B against AB5075 wild-type and its transposon mutants were examined. Logarithmic-phase broth cultures (in CaMHB) of respective bacterial isolates were centrifuged at 3,220 × *g* for 20 min to pellet bacteria. Pellets were subsequently resuspended in pre-warmed RPMI 1640 media supplemented with 25 mM HEPES and 10% heat-inactivated FBS to yield an inoculum of ~10^8^ CFU/mL, followed by polymyxin B treatment at 4 mg/L. Treated cultures were incubated at 37°C with shaking at 200 rpm. At baseline (0 h) and 1, 5, and 24 h post polymyxin B treatment, viable bacteria were quantified by manually plating 25 μL of appropriately diluted bacterial suspension onto nutrient agar followed by incubation at 37°C for 16–18 h prior to colony counting.

To examine the interacting growth of AB5075 WT and candidate transposon mutants following THP-1-dMs infection and polymyxin B treatment, THP-1-dMs were firstly infected at MOI 1,000 on 24-well plates and incubated at 37°C (5% CO_2_). At 3 h post infection, non-interacting extracellular bacteria were discarded and the cell monolayer was washed twice with pre-warmed DPBS (1×) before being replaced with pre-warmed cell culture media containing 0.25 mg/L polymyxin B. The experimental culture was further incubated at 37°C (5% CO_2_). At 1, 5, and 21 h post polymyxin B treatment, cell monolayers were washed twice with ice cold DPBS (1×) to remove non-interacting extracellular bacteria and THP-1-dMs were lysed in 1% Triton X-100 for 1 h on ice. The lysates containing the released interacting bacteria from infected THP-1-dMs were centrifuged at 10,000 × *g* for 10 min to pellet bacterial cells and remove the residual Triton X-100. The bacterial pellet was resuspended in 0.9% NaCl solution and 25 μL of bacterial suspension was spot-plated on NA following appropriate serial dilution, and then incubated at 37°C for 16–18 h prior to colony counting.

### Time-lapse live-cell imaging

Logarithmic-phase cultures of AB5075 wild-type and *spoT* mutant were firstly pelleted at 3,220 × *g* for 20 min and resuspended in RPMI 1640 media supplemented with HEPES and 10% heat-inactivated FBS. To ensure sufficient and non-overcrowding of bacterial cells throughout the 24-h continuous imaging, bacterial cell density was adjusted accordingly by examination on EVE Cell Counting Slides using a Leica DMi8 Inverted Microscope (Leica Microsystems) at 20× magnification. Images were taken at baseline (0 h) using untreated controls. Additionally, 40 μL of the adjusted bacterial cultures were added to respective treatment wells of μ-Slide VI (IBIDI) pre-loaded with 60 μL of media containing no polymyxin B (0 mg/L) or polymyxin B at 0.25 mg/L (1× minimum inhibitory concentration [MIC]) or 0.5 mg/L (2×MIC). The treatment slide was incubated on the microscope stage at 37°C. Using 20× magnification, twelve spots per treatment group were marked and traced, and images at all spots were collected every 10 min for 24 h. The time-lapse videos for each treatment group are attached in **S1–S6 Videos**. For analysis of bacterial cell size changes over time, images of the same spot collected every 1 h for 24 h were examined using ImageJ [96].

### Mouse bacteremia model

An immunocompetent mouse bacteremia model was employed to examine the survivability of *spoT* mutants and AB5075 wild-type, and polymyxin B efficacy *in vivo* (Monash University, Clayton, Victoria, Australia). Female Swiss mice (8-week-old, weight of 24–32 g) were housed with food and water available *ad libitum* and were used in the immunocompetent mouse bacteremia model (Monash University, Clayton, Victoria, Australia). Mice were randomly assigned to different experimental groups. Early logarithmic-phase cultures (100 μL) of AB5075 wild-type or *spoT* transposon mutants were intravenously administered into each female Swiss mouse to establish bloodstream infection (bacterial inoculum of 1×10^7^, 10^8^ or 10^9^ CFU/mouse). At 2 h after inoculation, polymyxin B was administered intravenously at 4 mg/kg to the treatment group. Mice were euthanized at 0 h and 4 h post polymyxin B treatment using 100% carbon dioxide inhalation, and blood was collected using cardiac puncture. Mouse blood was serially diluted with 0.9% saline and spirally plated on nutrient agar plates to determine bacterial load in blood (CFU/mL). At least three independent biological replicates per experimental group were examined in this study. All animal experiments were approved by the Monash University Animal Ethics Committee and performed in compliance with the Australian Code of Practice for the Care and Use of Animals for Scientific Purposes.

### Quantification and statistical analysis

Bacterial viable count in the *in vitro* time-kill assay and THP-1-dMs infection study were quantified using spot assay on nutrient agar plate (25 μL per spot; 40 CFU/mL as the limit of detection) and statistically analyzed using two-way ANOVA followed by Tukey’s HSD post-hoc test on GraphPad Prism 9.1.0. In the mouse bacteremia study, bacterial load in blood was quantified by spirally spreading on nutrient agar plates (50 μL; 20 CFU/mL as the limit of detection). Multiple unpaired *t*-tests were performed in GraphPad Prism 9.1.0 to analyze the *in vivo* bacterial count, with p-value <0.05 defined as statistically significant. The sample size used in each experiment is specified in the methodology sections and figure legends.

## Supporting information

S1 Fig Experimental workflow of proteomic analysis of *A*. *baumannii* and THP-1-dMs. (TIF)

S2 Fig Distinct global proteomic changes in *A*. *baumannii* and THP-1-dMs following polymyxin treatment. **A**) PCA score plot of *A*. *baumannii* proteomic profiles showing the relatedness of the dataset within and across the experimental groups of untreated AB5075 (i.e., controls; AB), polymyxin B treated AB5075 (AB + PMB), interacting AB5075 from infected THP-1-dMs (AB + THP-1-dMs), and interacting AB5075 from polymyxin B treated THP-1-dMs infection (AB + THP-1-dMs + PMB). **B**) Venn diagram showing common and unique sets of differentially expressed proteins of *A*. *baumannii* (log_2_FC >1 or <-1, FDR <0.05) between different comparison groups. **C**) PCA score plot of THP-1-dMs proteomic profiles showing the relatedness of the dataset within and across the experimental groups of untreated THP-1-dMs (i.e., controls; THP-1-dMs), AB5075-infected THP-1-dMs (AB + THP-1-dMs), polymyxin B treated THP-1-dMs (THP-1-dMs + PMB), and polymyxin B treated and AB5075-infected THP-1-dMs (AB + THP-1-dMs + PMB). **D**) Venn diagram showing common and unique sets of differentially expressed proteins of THP-1-dMs (log_2_FC >1 or <-1, FDR <0.05) between different comparison groups. Three biological replicates were employed in each experimental group.
(TIF)

S3 Fig *A*. *baumannii* infection predominantly enriches proteins in coagulation cascade associated pathways in THP-1-dMs. Volcano plot generated using WebGestalt showing the enriched KEGG and Reactome pathways (Benjamini-Hochberg FDR <0.05) in THP-1-dMs at 4 h post infection with AB5075 in the absence of polymyxin B.
(TIF)

S1 Table List of all DEPs in interacting *A*. *baumannii* following infection of THP-1-dMs (AB + THP-1-dMs) compared to the untreated bacteria control (log_2_FC >1 or <-1, FDR <0.05). DEPs, differentially expressed proteins; AB, wild-type *Acinetobacter baumannii* AB5075; PMB, polymyxin B; THP-1-dMs, macrophage-like THP-1 cells.
(XLSX)

S2 Table List of all DEPs in polymyxin B treated *A*. *baumannii* (AB + PMB) compared to the untreated bacteria control (log_2_FC >1 or <-1, FDR <0.05). DEPs, differentially expressed proteins; AB, wild-type *Acinetobacter baumannii* AB5075; PMB, polymyxin B; THP-1-dMs, macrophage-like THP-1 cells.
(XLSX)

S3 Table List of all DEPs in interacting *A*. *baumannii* following polymyxin B treated THP-1-dMs infection (AB + THP-1-dMs + PMB) compared to the untreated bacteria control (log_2_FC >1 or <-1, FDR <0.05). DEPs, differentially expressed proteins; AB, wild-type *Acinetobacter baumannii* AB5075; PMB, polymyxin B; THP-1-dMs, macrophage-like THP-1 cells.
(XLSX)

S4 Table List of AB5075-UW T26 transposon mutants used in functional studies. (XLSX)

S1 Video 24-h time-lapse live-cell imaging of *A*. *baumannii* AB5075 wild-type (WT) in the absence of polymyxin B (PMB) treatment. 20× magnification; video speed at 7 frames per second (fps).
(AVI)

S2 Video 24-h time-lapse live-cell imaging of *A*. *baumannii* AB5075 *spoT* transposon mutant in the absence of polymyxin B (PMB) treatment. 20× magnification; video speed at 7 frames per second (fps).
(AVI)

S3 Video 24-h time-lapse live-cell imaging of *A*. *baumannii* AB5075 wild-type (WT) in the presence of 1×MIC polymyxin B (PMB; 0.25 mg/L) treatment. 20× magnification; video speed at 7 frames per second (fps).
(AVI)

S4 Video 24-h time-lapse live-cell imaging of *A*. *baumannii* AB5075 *spoT* transposon mutant in the presence of 1×MIC polymyxin B (PMB; 0.25 mg/L) treatment. 20× magnification; video speed at 7 frames per second (fps).
(AVI)

S5 Video 24-h time-lapse live-cell imaging of *A*. *baumannii* AB5075 wild-type (WT) in the presence of 2×MIC polymyxin B (PMB; 0.5 mg/L) treatment. 20× magnification; video speed at 7 frames per second (fps).
(AVI)

S6 Video 24-h time-lapse live-cell imaging of *A*. *baumannii* AB5075 *spoT* transposon mutant in the presence of 2×MIC polymyxin B (PMB; 0.5 mg/L) treatment. 20× magnification; video speed at 7 frames per second (fps).
(AVI)
