# Supplementary figures and images for: Correlative proteomics identify the key roles of stress tolerance strategies in Acinetobacter baumannii in response to polymyxin and human macrophages
Source: PLoS Pathog. 2022 Mar 1;18(3):e1010308. doi: 10.1371/journal.ppat.1010308 (PMC8887720; doi:10.1371/journal.ppat.1010308)

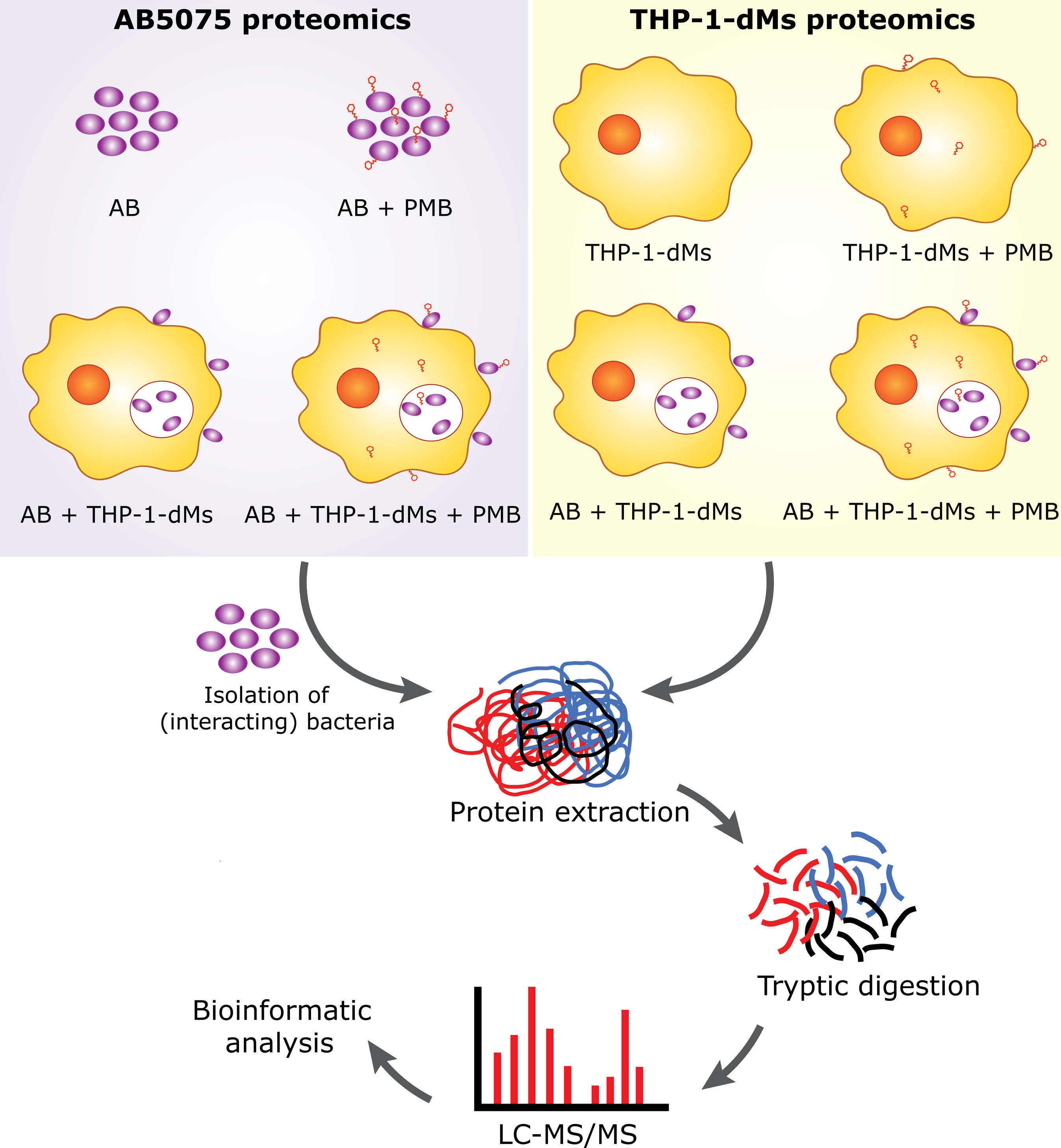

Supplement: S1 Fig — (TIF) [file ppat.1010308.s001.tif]

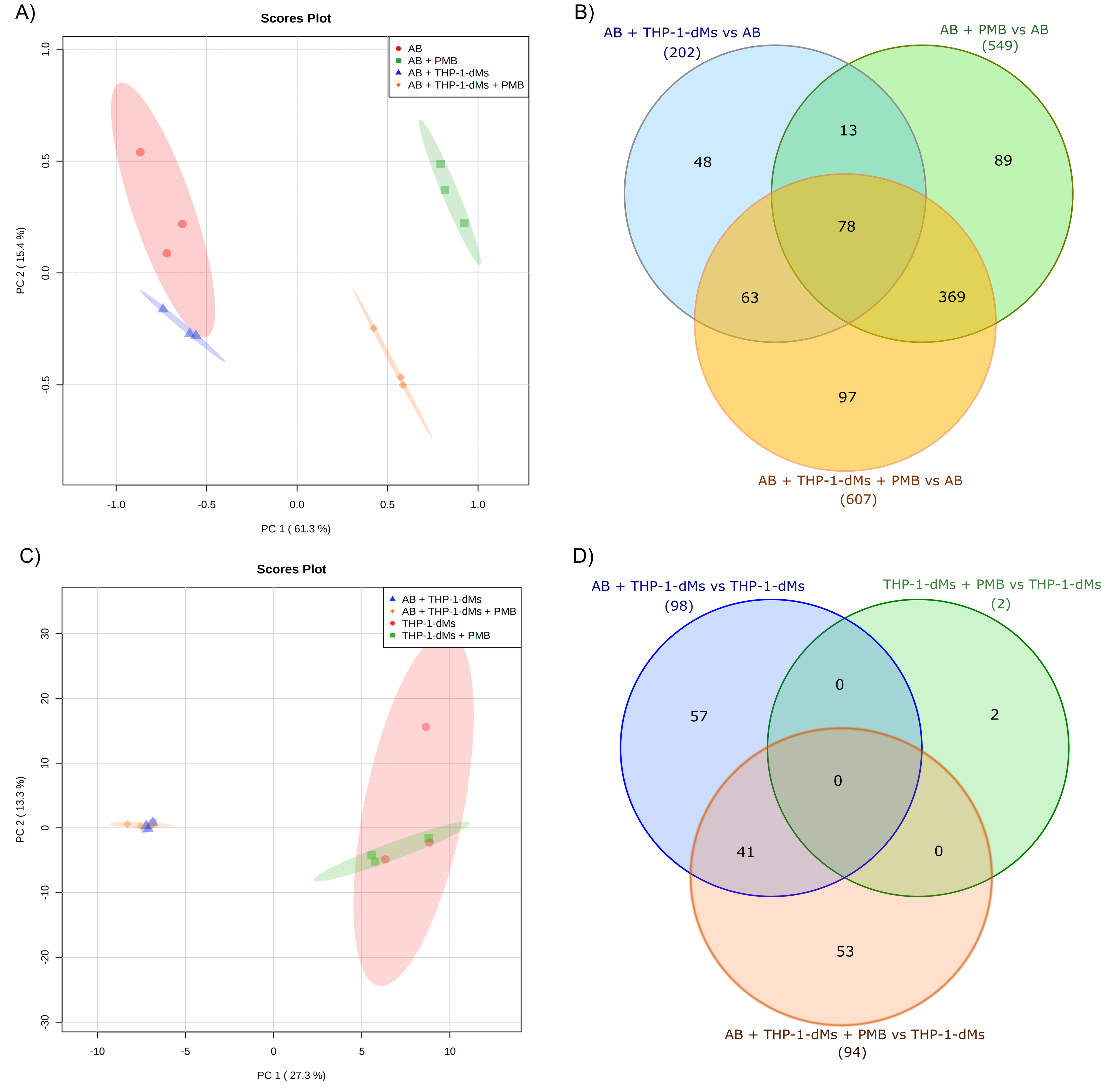

Supplement: S2 Fig — A) PCA score plot of A. baumannii proteomic profiles showing the relatedness of the dataset within and across the experimental groups of untreated AB5075 (i.e., controls; AB), polymyxin B treated AB5075 (AB + PMB), interacting AB5075 from infected THP-1-dMs (AB + THP-1-dMs), and interacting AB5075 from polymyxin B treated THP-1-dMs infection (AB + THP-1-dMs + PMB). B) Venn diagram showing common and unique sets of differentially expressed proteins of A. baumannii (log2FC >1 or <-1, FDR <0.05) between different comparison groups. C) PCA score plot of THP-1-dMs proteomic profiles showing the relatedness of the dataset within and across the experimental groups of untreated THP-1-dMs (i.e., controls; THP-1-dMs), AB5075-infected THP-1-dMs (AB + THP-1-dMs), polymyxin B treated THP-1-dMs (THP-1-dMs + PMB), and polymyxin B treated and AB5075-infected THP-1-dMs (AB + THP-1-dMs + PMB). D) Venn diagram showing common and unique sets of differentially expressed proteins of THP-1-dMs (log2FC >1 or <-1, FDR <0.05) between different comparison groups. Three biological replicates were employed in each experimental group. (TIF) [file ppat.1010308.s002.tif]

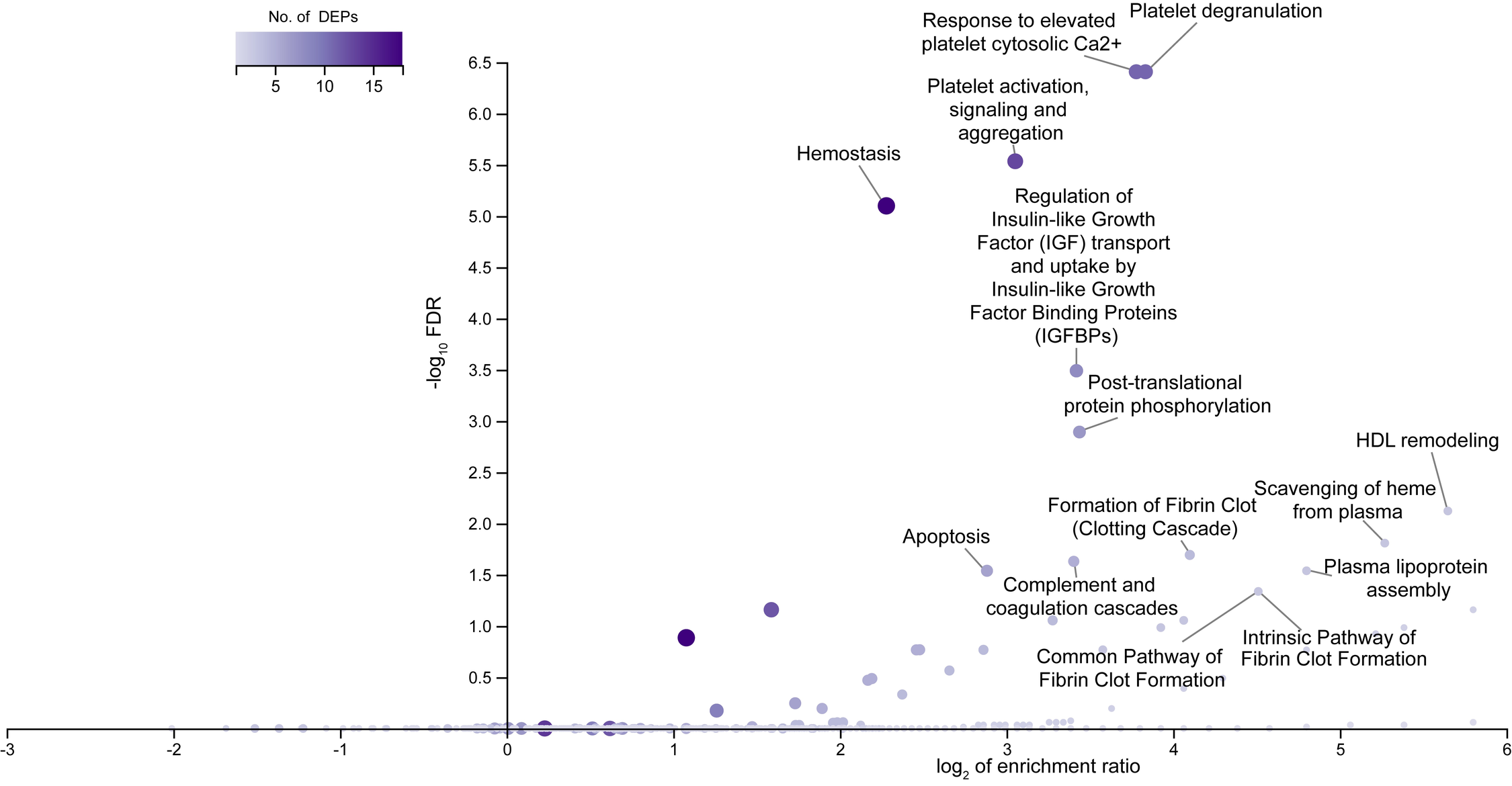

Supplement: S3 Fig — Volcano plot generated using WebGestalt showing the enriched KEGG and Reactome pathways (Benjamini-Hochberg FDR <0.05) in THP-1-dMs at 4 h post infection with AB5075 in the absence of polymyxin B. (TIF) [file ppat.1010308.s003.tif]
